# Relative abundance of mature myostatin rather than total myostatin is negatively associated with bone mineral density in Chinese

**DOI:** 10.1111/jcmm.13438

**Published:** 2017-12-16

**Authors:** Long‐Fei Wu, Dong‐Cheng Zhu, Bing‐Hua Wang, Yi‐Hua Lu, Pei He, Yun‐Hong Zhang, Hong‐Qin Gao, Xiao‐Wei Zhu, Wei Xia, Hong Zhu, Xing‐Bo Mo, Xin Lu, Lei Zhang, Yong‐Hong Zhang, Fei‐Yan Deng, Shu‐Feng Lei

**Affiliations:** ^1^ Center for Genetic Epidemiology and Genomics School of Public Health Soochow University Suzhou Jiangsu China; ^2^ Jiangsu Key Laboratory of Preventive and Translational Medicine for Geriatric Diseases Soochow University Suzhou Jiangsu China; ^3^ Department of Orthopedics Sihong People's Hospital Suqian Jiangsu China; ^4^ Disease Prevention and Control Center of Suzhou high Tech Zone Suzhou Jiangsu China; ^5^ Shishan Community Health Service Center Suzhou High Tech Zone Suzhou Jiangsu China

**Keywords:** bone‐muscle interactions, osteoporosis, osteoblasts, DXA, myostatin

## Abstract

Myostatin is mainly secreted by skeletal muscle and negatively regulates skeletal muscle growth. However, the roles of myostatin on bone metabolism are still largely unknown. Here, we recruited two large populations containing 6308 elderly Chinese and conducted comprehensive statistical analyses to evaluate the associations among lean body mass (LBM), plasma myostatin, and bone mineral density (BMD). Our data revealed that total myostatin in plasma was mainly determined by LBM. The relative abundance of mature myostatin (mature/total) was significantly lower in high *versus* low BMD subjects. Moreover, the relative abundance of mature myostatin was positively correlated with bone resorption marker. Finally, we carried out *in vitro* experiments and found that myostatin has inhibitory effects on the proliferation and differentiation of human osteoprogenitor cells. Taken together, our results have demonstrated that the relative abundance of mature myostatin in plasma is negatively associated with BMD, and the underlying functional mechanism for the association is most likely through inhibiting osteoblastogenesis and promoting osteoclastogenesis.

## Introduction

Myostatin is mainly produced by skeletal muscles and served as a negative regulator of muscle growth 1, 2. Muscle‐derived myostatin is released into plasma in a form of precursor protein (pro‐myostatin), which can be cleaved into mature myostatin by BMP‐1/tolloid family of metalloproteinases 3. Besides, this precursor protein can also be activated by hydrochloric acid treatment *in vitro* 4. Only, the mature myostatin can activate downstream Smad 2/3 signalling pathway 5, 6. In addition, the functions of myostatin are inhibited by multiple extracellular molecular proteins, such as follistatin, which can interact with myostatin and prevent their binding to cell surface receptors 7, 8.

Muscle and bone are two closely related organs in musculoskeletal system 9. Recent studies have shown that myostatin is functionally relevant to bone remodelling 10, 11, 12, 13, 14. Myostatin knockout mice have greater femoral bone mineral density, and the underlying mechanism is mainly due to the increased mechanical load 15. Moreover, myostatin plays essential roles in bone regeneration by inhibiting the recruitment and proliferation of osteoprogenitor cells in the fracture blastema 11. Importantly, myostatin is highly expressed in the synovial tissues of rheumatoid arthritis (RA) subjects, and the inhibition of its activity can prevent bone destruction by regulating osteoclastogenesis 16. However, these conclusions from animal models are difficult to be extended to humans. Therefore, it is necessary to examine whether myostatin is related to bone metabolism in human population, herein in Chinese.

By adopting an extreme sampling scheme and multiple‐stage analyses, we tested the association of plasma myostatin with BMD, bone turnover indexes, as well as a few other metabolism indexes in two populations containing 6308 elderly Chinese. Subsequently, we examined *in vitro* effects of myostatin on osteoblastogenesis (osteoblast proliferation and differentiation). Our data suggested that relative mature myostatin in plasma is negatively associated with BMD in Chinese, and the underlying functional mechanism for the association is most likely through inhibiting osteoblastogenesis and promoting osteoclastogenesis. These findings shed new lights on the functions of myostatin on bone metabolism, which would improve our understanding of the myostatin‐mediated muscle–bone interaction.

## Materials and Methods

### Samples

**Figure 1:**
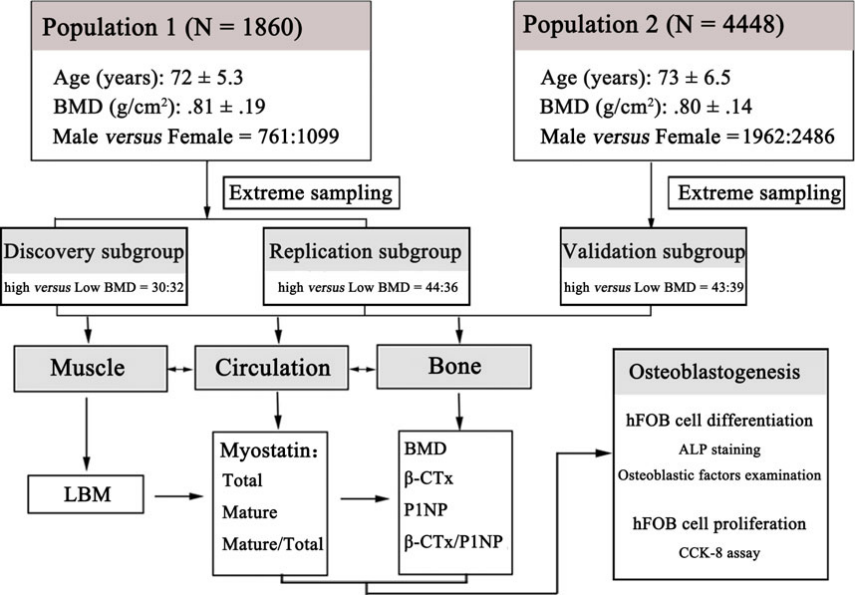
Overview of the flow chart for this study. The whole study is divided into three parts as follows: subject recruitment and extreme sampling; data acquisition and comprehensive analyses at distinct aspects (muscle, circulation and bone); and *in vitro* experiments to investigate the function of myostatin on proliferation and differentiation of osteoprogenitor cells.

The flow chart of the study is presented in Figure 1. We first recruited two independent populations (Population 1, *N* = 1860; Population 2, *N* = 4448) of elderly Chinese. Next, we adopted extreme sampling strategy and selected three subgroups according to hip BMD. Subsequently, we measured myostatin levels in plasma and conducted comprehensive analyses at distinct aspects (muscle, circulation and bone). Lastly, we investigated the function of myostatin on the proliferation and differentiation of osteoprogenitor cells.

We sorted the subjects according to BMD. Then, subjects with extremely high and low BMD were selected from top and bottom 10%, respectively. From the Population 1, we randomly selected two extreme subgroups for discovery (*N* = 62, high *versus* low BMD = 30:32) and replication (*N* = 80, high *versus* low BMD = 44:36) purposes, respectively. The validation subgroup (*N* = 82, high *versus* low BMD = 43:39) was selected from the Population 2.

Both populations 1 and 2 were from our ongoing Osteoporosis Prevention Project (OPP), with major goal to identify osteoporosis risk biomarkers. All participants were Chinese Han ethnic living in the eastern China in Suzhou, Jiangsu. Information such as age, medication history and disease history was obtained *via* questionnaire. Written informed consent documents were obtained from all study participants before entering the study. The study was approved by Institutional Research Ethic Board at the Soochow University.

To minimize potential confounding effects on bone metabolism, strict criteria were adopted excluding chronic disorders involving vital organs (*e.g*. heart, lung, liver, kidney and brain), serious metabolic diseases (*e.g*. diabetes, hypo‐ or hyperparathyroidism and hyperthyroidism), other skeletal diseases (*e.g*. Paget disease and rheumatoid arthritis), cancers (*e.g*. prostate, ovary and breast cancer), chronic use of drugs affecting bone metabolism and myostatin levels (*e.g*. corticosteroid therapy and anticonvulsant drugs) and malnutrition conditions (*e.g*. chronic diarrhoea and chronic ulcerative colitis).

### BMD measurement

For each subject, areal BMD (g/cm^2^) at femoral neck, trochanter and intertrochanter was measured by dual‐energy X‐ray absorptiometry (DXA) with Hologic densitometers (Hologic Inc., Waltham, MA, USA). Total hip BMD value was established by the three measured regions. The precision of BMD is expressed as the root‐mean‐square per cent coefficient of variation (RMS‐CV) to determine the least significant change in bone density 17. We tested thirty subjects for three times to assess the precision with the RMS‐CV is 2.49%.

### Myostatin qualification

For each subject, peripheral blood was drawn in the early morning after the examinees fasted overnight. Plasma was separated and kept frozen at −80°C until the measurement. Plasma total myostatin includes both mature myostatin and precursor protein (pro‐myostatin). Total myostatin levels were measured by ELISA (GDF‐8/Myostatin Immunoassay, R&D systems Inc., Minneapolis, USA) according to the manufacture's instruction with acid activation, which includes the abundance of both precursor protein and mature myostatin 4. Specifically, plasma samples were incubated at room temperature with 1M HCl for 10 min. to remove the pro‐peptide from pro‐myostatin. After activation, the form of pro‐myostatin in plasma would be converted to immunoreactive form. Thus, total myostatin levels we measured in plasma after acid activation were composed of mature and pro‐myostatin. Mature myostatin levels were determined by ELISA without acid activation. In the study, we used three myostatin parameters in the following analysis including total myostatin, mature myostatin and the relative abundance of mature myostatin (mature/total myostatin). Other bone turnover markers, such as β‐CTx and P1NP, were determined by ELISA kit (Feikang Biotec, Guangzhou, China). The concentrations of plasma glucose, total cholesterol and triglycerides were measured by an auto‐analyser (Olympus, Tokyo, Japan).

### Estimation of LBM

As the LBM measured by DXA whole body scan is unavailable, we calculated the LBM using the mathematical formula, which was estimated from height and weight 18. Moreover, the correlation coefficients between the calculated LBM and the measured LBM by DXA for males and females were 0.913 and 0.872, respectively (*P* < 0.001), implying that this formula is accurate in evaluating LBM. The used formula is as follows:

For men: LBM = (0.328 * *W*) + (0.339 * *H*) − 29.533

For women: LBM = (0.295 * *W*) + (0.418 * *H*) − 43.293

*W* is body weight in kilograms (kg), and *H* is body height in centimetres (cm).

### Cell culture

The human foetal osteoblastic 1.19 cell line (hFOB) was purchased from the Institute of Cell Bank/Institutes for Biological Sciences (Shanghai, China). For *in vitro* proliferation, hFOB cells were maintained at 33.5°C in the complete medium consisting of 1:1 DMEM/Ham's F‐12 medium (Life Technologies, Grand Island, NY, USA) without phenol red supplemented with 10% foetal bovine serum, 0.3 mg/ml G418 (Roche Diagnostics, Mannheim, Germany) and penicillin/streptomycin (Life Technologies). For *in vitro* osteogenic differentiation, hFOB cell line was maintained at 37°C in complete medium supplemented with 10 mM M‐glycerol phosphate, 0.25 mM ascorbic acid and 0.1 μM dexamethasone (all from Sigma‐Aldrich Corp., St. Louis, MO, USA).

### Real‐time quantitative PCR

Total RNA of hFOB cells were extracted with TRIzol (Life Technologies). cDNA was synthesized using 1 μg RNA with a reverse transcription kit (Promega, Madison, WI, USA). Amplification reactions were performed by a Light Cycler PRISM 7500 (Applied Biosystems, Foster City, CA, USA) using the primers of runt‐related transcription factor/2 (Runx2), osteocalcin (OC), alkaline phosphatase (ALP) and glyceraldehyde‐3‐phosphate dehydrogenase (GAPDH). The mRNA expression levels were normalized to GAPDH, and the relative quantification was calculated using the 2^−ΔΔCT^ method.

### ALP staining assay

hFOB cells were incubated in the osteogenic medium, supplemented with various concentrations of recombinant myostatin (R&D Systems), which was a mature form of myostatin. ALP staining was performed *via* the TRACP & ALP double‐stain Kit (TaKaRa Bio, Shiga, Japan). For ALP staining, cells were rinsed twice with PBS and then fixed in fixation buffer. After washed by distilled water, cells were stained with ALP substrate. The experiments were performed in duplicate for each condition.

### Cell proliferation assay

hFOB cells were seeded into 96‐well plate with a density of 0.6 × 10^3^ cells per well and cultured at the complete medium at 33.5°C for 24 hrs. The medium was then replaced by fresh medium treated with recombinant myostatin at the beginning of the assay with different concentrations: 0, 10, 50 and 100 ng/ml. At selected time interval (1, 2, 3, 4 and 5 days), the hFOB cells proliferation was measured by the CCK‐8 assay kit (Dojindo Molecular Technologies, Gaithersburg, Japan) according to the manufacturer's instructions. Briefly, 10 μl CCK‐8 solution was added to each well and then incubated for 2 hrs in 37°C. The absorbance at 450 nm was determined by microplate reader (BioTek, Wellesley, MA, USA).

### Statistical analysis

Continuous data were expressed as mean ± S.D. Linear regression analysis, Student's *t*‐test, Pearson correlation analysis and Analysis of Covariance were performed to evaluate associations within the studied indexes. The *P* values from individual tests were combined using Fisher's method to quantify the overall evidence for association 19, 20. The used formula is as follows:

$$X_{2k}^{2}=-2\sum_{i=1}^{\text{k}}{\text{log}}_{e}\left(p_{i}\right)$$

where 2*k* is the degree of freedom of the *X* statistic, and *k* is the number of tests being combined. All statistical analyses were performed with the SPSS 16.0. Statistical significance was defined as *P* < 0.05.

## Results

### Basic characteristics of the three selected subgroups

In this study, three subgroups were selected from Population 1 (Discovery subgroup, *N* = 62; Replication subgroup, *N* = 80) and Population 2 (Validation subgroup, *N* = 82) according to BMD. As shown in Table 1, significant difference for BMD can be observed between high and low BMD subjects in the three subgroups. As expected, we found that bone turnover markers, such as β‐CTx and P1NP (except in replication subgroup), were lower in high *versus* low BMD subjects. However, there was no difference in total cholesterol, blood glucose (except in replication subgroup) or triglycerides between high and low BMD subjects.

**Table 1:** Basic characteristics of the three subgroups from population 1 and population 2

	Discovery subgroup			Replication subgroup			Validation subgroup		
	High BMD (*n* = 30)	Low BMD (*n* = 32)	*P* value	High BMD (*n* = 44)	Low BMD (*n* = 36)	*P* value	High BMD (*n* = 43)	Low BMD (*n* = 39)	*P* value
Clinical									
Age (year)	69.3 ± 2.9	71.0 ± 2.6	**0.029**	70.8 ± 2.8	69.4 ± 2.6	**0.027**	68.2 ± 3.1	69.5 ± 3.0	**0.062**
Height (cm)	160.4 ± 9.3	158.3 ± 8.1	0.362	160.8 ± 7.8	157.4 ± 8.8	0.074	159.3 ± 25.3	153 ± 25.7	0.318
Weight (kg)	66.8 ± 11.1	54.5 ± 8.8	**<0.001**	68.9 ± 8.4	55.2 ± 9.3	**<0.001**	71.3 ± 15.4	52.9 ± 13.7	**<0.001**
LBM	44.1 ± 6.6	39.5 ± 5.3	**0.005**	45.8 ± 6.0	40.2 ± 6.9	**<0.00**1	47.8 ± 6.4	43.3 ± 4.2	**0.001**
Bone									
BMD (g/cm^2^)	1.06 ± 0.07	0.59 ± 0.09	**<0.001**	1.05 ± 0.09	0.63 ± 0.09	**<0.001**	1.12 ± 0.11	0.58 ± 0.08	**<0.001**
P1NP (ng/ml)	20.6 ± 12.7	38.0 ± 14.6	**0.001**	39.4 ± 13.6	42.9 ± 18.3	0.323	37.9 ± 15.3	49.7 ± 17.0	**0.003**
β‐CTx (ng/ml)	0.17 ± 0.14	0.35 ± 0.21	**0.002**	0.23 ± 0.17	0.35 ± 0.19	**0.003**	0.20 ± 0.18	0.33 ± 0.30	**0.021**
Metabolism index									
Blood glucose (mmol/l)	5.79 ± 0.86	6.17 ± 1.71	0.284	6.08 ± 1.48	5.40 ± 0.52	**0.010**	6.22 ± 1.43	5.61 ± 1.3	0.055
Total cholesterol (mmol/l)	3.77 ± 0.71	3.89 ± 0.80	0.523	3.81 ± 0.59	3.86 ± 0.71	0.755	4.81 ± 1.0	4.71 ± 0.84	0.676
Triglyceride (mmol/l)	1.95 ± 1.17	1.55 ± 0.99	0.153	1.59 ± 0.80	1.41 ± 0.69	0.308	1.28 ± 0.57	1.12 ± 0.66	0.236

Data are presented as mean ± S.D. Bold values indicate statistical significance.

*P* value represents for comparison of high BMD *versus* low BMD, and comparisons are not controlled for any variables.

BMD, bone mineral density; LBM, lean body mass; P1NP, pro‐collagen type I amino‐terminal pro‐peptide; β‐CTx, β‐isomerization of the C‐terminal telopeptide of type I collagen.

### Positive association of plasma myostatin and LBM

**Figure 2:**
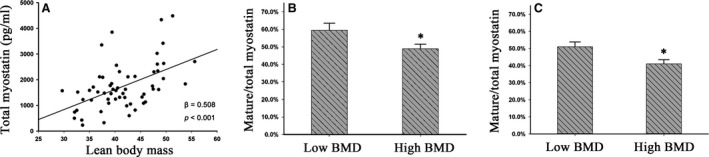
Association of plasma myostatin with LBM and BMD. (**A**) Association between total plasma myostatin and LBM; Linear regression analysis was conducted, and gender and age were used as two covariates; β, regression coefficients. (**B** and **C**) Association between mature/total myostatin and BMD in the replication (**B**) and validation subgroup (**C**); data were presented as mean ± S.D. Statistical differences between the two groups were determined by the student's *t*‐test. *P < 0.05.

Considering myostatin in plasma is mainly secreted by skeletal muscles, we first tested the relationship between LBM and myostatin levels in plasma. As expected, positive association of total myostatin and LBM was observed after adjustment for age and gender (β = 0.51, *P* < 0.001; Fig. 2A). Consistently, such correlation was detected in the replication (β = 0.34, *P* < 0.001) and validation subgroups (β = 0.29, *P* = 0.012). These results supported the concept that total myostatin in plasma is derived from muscle and mainly determined by LBM.

### The difference in plasma myostatin levels between high and low BMD group

To further investigate the effect of myostatin on BMD, we first compared total myostatin levels in subjects with high BMD *versus* low BMD. The concentration of total myostatin in plasma was positively associated with BMD in the discovery subgroup (*P* = 0.044), replication subgroup (*P* = 0.047) and validation subgroup (*P* = 0.018). After adjusting LBM, the positive correlation between BMD and total myostatin in plasma disappeared in three subgroups, suggesting that positive correlation of total myostatin and BMD is mainly dependent on LBM.

Skeletal muscle‐secreted myostatin remains inactive in a form of precursor protein in blood, and only mature myostatin could activate downstream signalling. Therefore, we further measured mature myostatin levels and calculated relative abundance of mature myostatin (mature/total myostatin) in the replication subgroup. The relative abundance of mature myostatin in plasma was about 54 ± 2.4%. For mature myostatin, the difference between high and low BMD subjects was insignificant (1.69 ± 0.08 *versus* 1.79 ± 0.11 ng/ml). However, the relative abundance of myostatin levels was significantly decreased in high BMD individuals (49.0 ± 4.1%) when compared with low BMD individuals (59.5 ± 2.5%, *P* = 0.021; Fig. 2B). To further confirm our results, we examined mature and total myostatin in the independent validation subgroup (*N* = 82). Likewise, the relative abundance of mature myostatin was significantly decreased in high BMD subjects compared to low BMD subjects (41.0 ± 2.4% *versus* 51.0 ± 2.8%, *P* = 0.014; Fig. 2C). Moreover, we combined *P* values from both replication and validation subgroups using Fisher's combined P method 20 and observed that the association became much more significant (*P* < 0.005), warranting that the relative abundance of mature myostatin is negatively correlated with BMD in elderly Chinese population.

### Association of plasma myostatin with other metabolic parameters

To further test the underlying mechanism for the associations between plasma myostatin and BMD, we analysed the relationships between plasma myostatin and other metabolic factors including commonly used bone turnover markers (*e.g*. P1NP and β‐CTx) and metabolism parameters (*e.g*. triglycerides and cholesterol; Table 2). Correlation analysis revealed that relative abundance of mature myostatin was positively associated with β‐CTx in replication subgroup (*r* = 0.27, *P* = 0.035), and suggestive correlation was observed in validation subgroup (*r* = 0.23, *P* = 0.092). Among lipid metabolism parameters, only triglycerides levels were correlated with mature/total myostatin in validation subgroup. Collectively, these results above further implied the relationship between myostatin and bone metabolism.

**Table 2:** Pearson's correlation of myostatin with studied parameters in replication and validation subgroups

Variable	Replication subgroup			Validation subgroup		
	Total	Mature	Mature/Total	Total	Mature	Mature/Total
Age	0.02(0.88)	0.15(0.23)	−0.07(0.58)	0.215(0.57)	0.16(0.17)	0.04(0.72)
LBM	**0.33**(**<0.01**)	0.14(0.28)	−0.24(0.06)	**0.24**(**0.01**)	0.17(0.14)	−0.11(0.33)
Metabolism index						
Plasma glucose	−0.49(0.70)	−0.22(0.07)	0.12(0.36)	−0.07(0.54)	−0.04(0.71)	0.05(0.68)
Triglycerides	−0.09(0.48)	−0.06(0.65)	−0.07(0.61)	−0.19(0.08)	−0.05(0.68)	**0.31**(**0.01**)
Plasma cholesterol	−0.02(0.87)	−0.13(0.29)	−0.02(0.91)	−0.14(0.21)	−0.06(0.62)	0.15(0.16)
Bone turnover						
β‐CTx	−0.19(0.14)	−0.06(0.61)	**0.27**(**0.04**)	−0.04(0.72)	−0.10(0.36)	0.23(0.09)
P1NP	−0.03(0.80)	−0.13(0.27)	0.11(0.41)	−0.08(0.47)	−0.08(0.50)	0.13(0.37)

Data are presented as coefficients (Pearson correlation *P* value). Bold values indicate statistical significance.

### The effect of myostatin on hFOB cells differentiation and proliferation

**Figure 3:**
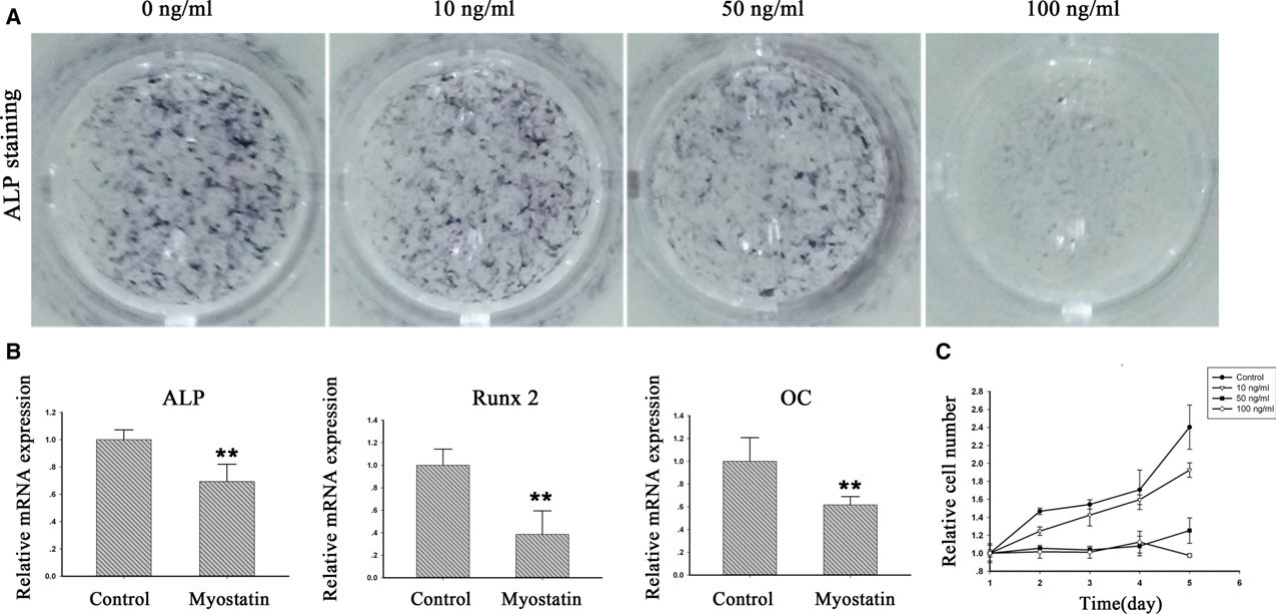
Myostatin inhibits hFOBs differentiation and proliferation *in vitro*. (**A**) Representative images of ALP staining in hFOBs after treatment with different concentrations of mature myostatin (0, 10, 50 and 100 ng/ml) in osteogenic medium for 7 days. (**B**) Expression level of bone differentiation markers (*e.g*. ALP, Runx2 and OC) in hFOBs treated with or without mature myostatin for 3 days. The mRNA expression levels were examined by RT‐PCR (*N* = 3 separate experiments), using GAPDH as internal control. All data were presented as mean ± S.D., from three independent experiments.**P < 0.01. (**C**) Time course changes in cell numbers under treatment with different concentration of myostatin (0, 10, 50 and 100 ng/ml) in complete medium. After incubation with CCK‐8 solution, cell numbers were measured *via* the absorbance at 450 nm by microplate reader. All data were presented as mean ± S.D.

Previous studies have reported that the myostatin functions to promote the process of osteoclastogenesis. In this study, we focused on its effect on the other side of bone turnover, that is, osteoblastogenesis. We used human foetal osteoblastic cell line as a model system to study the effect of myostatin on its differentiation and proliferation *in vitro*. Considering pro‐myostatin has no biological activity, we just used mature myostatin with gradually increased concentrations (0, 10, 50 and 100 ng/ml) to monitor changes of the relative abundance of mature myostatin in human plasma. As shown in Figure 3A, hFOB cells cultured with osteogenic medium for 7 days showed increased ALP staining. However, ALP staining was decreased when hFOBs were treated with myostatin, indicating its inhibitory effect on hFOB cells differentiation. Next, we examined the effects of myostatin on the expression of osteoblastic factors and found the expression of osteoblast‐specific genes, such as ALP, Runx2 and OC, was significantly reduced when cultured with myostatin (Fig. 3B). Subsequently, we investigated the effects of myostatin on hFOBs proliferation. The hFOBs were treated with different concentrations of myostatin in non‐differentiation medium. The CCK‐8 assay demonstrated that myostatin could inhibit hFOBs proliferation in a dose‐dependent manner (Fig. 3C). Thus, these data *in vitro* supported a hypothesis that myostatin may regulate osteoblastogenes through inhibiting osteoprogenitor differentiation and proliferation, which consequently contribute to BMD variation in human.

## Discussion

This study is the first population‐based association study between BMD and different forms of plasma myostatin. The results showed that relative abundance of mature myostatin is negatively associated with BMD in Chinese. *In vitro* data revealed that myostatin with gradually increased concentrations may regulate bone metabolism by inhibiting osteoprogenitor cell differentiation and proliferation in a dose‐dependent manner. Combine with previous studies, we hypothesize that subjects with higher level of mature/total myostatin have increased bone resorption and decreased bone formation, and consequently leading to decreased BMD.

Previous studies usually examined the circulating total myostatin only and evaluated its correlation with diseases, for example, obesity, but not osteoporosis yet 21, 22, 23. This study represented the first efforts in measuring both precursor and mature forms of plasma myostatin simultaneously and testing their relationship with osteoporosis at distinct levels (muscle, circulation and bone) in human population. Our results indicated total myostatin was mainly determined by LBM, while relative mature myostatin was significantly and negatively associated with BMD. Interestingly, higher level of total myostatin was observed in subjects with high BMD. This seemingly counter‐intuitive result is probably due to the correlation between BMD and LBM 24. After adjusting LBM, total myostatin was no longer correlated with BMD. Therefore, such results further increased our understanding not only in the complex regulation of myostatin maturation, but also the functional mechanism underlying the interaction of muscle and bone.

This study revealed that the relative abundance of mature myostatin in plasma was negatively associated with BMD in Chinese. Probably, skeletal muscle‐derived myostatin undergoes complex post‐translational modification to guarantee the balance of mature/total myostatin in plasma. Once the balance is disrupted, higher ratio of mature/total myostatin probably leads to decreased BMD, while lower ratio of mature/total myostatin results in increased BMD. Notably, the ratio of mature/total myostatin instead of mature myostatin was positively correlated with bone turnover marker β‐CTx. Thus, the relative abundance of mature myostatin may serve as a better index to evaluate the association of myostatin and BMD *in vivo*.

**Figure 4:**
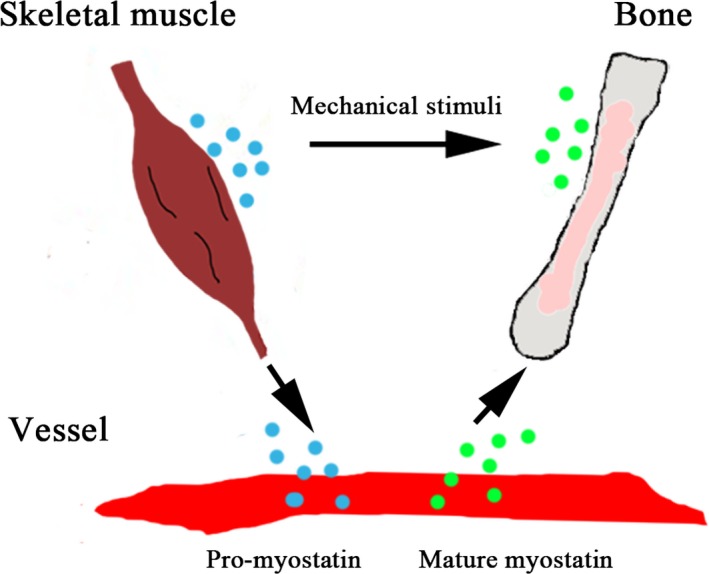
A proposed model of myostatin‐mediated muscle–bone interaction. Myostatin is mainly expressed in skeletal muscle and then secreted into extracellular and circulation. After post‐translational modifications, mature myostatin regulates bone metabolism by promoting bone resorption and inhibiting bone formation. Blue dot represents pro‐myostatin, and green dot represents mature myostatin.

Based on the present findings and previous evidence, we proposed a model of myostatin‐mediated muscle–bone interaction. As shown in Figure 4, pro‐myostatin is derived from muscle and is secreted into extracellular space and circulation, and then pro‐myostatin is converted into mature myostatin in plasma through complex post‐translational modifications. Bone homoeostasis can be tuned by the balance of myostatin secretion and maturation. LBM may determine the total myostatin levels in plasma. The relative abundance of mature myostatin may be correlated with downstream bone‐remodelling activation. These bone‐remodelling pathways include but not limit to those involved in human osteoblastogenesis and osteoclastogenesis. Future efforts are needed to validate and improve the model. For example, it's also necessary to disclose how myostatin homoeostasis is maintained in circulation, and how the broken balance influences bone metabolism.

Taken together, based on two populations with a total of 6308 elderly Chinese, circulating myostatin levels were examined in this study. Then, comprehensive analyses were conducted to evaluate the relationship of plasma myostatin with LBM and BMD. Collectively, our results revealed that total myostatin is determined by LBM, whereas the relative abundance of mature myostatin is negatively associated with BMD in elderly Chinese. The underlying functional mechanism for the association is most likely through inhibiting osteoblastogenesis and promoting osteoclastogenesis.

## Conflict of interests

The authors declare no conflict of interest.
